# Gender identity and symptoms of anxiety and depression and their relationship with sleep disorders among Polish adolescents during the Covid-19 pandemic and the outbreak of war in the Ukraine

**DOI:** 10.1192/j.eurpsy.2025.1479

**Published:** 2025-08-26

**Authors:** K. Badura-Brzoza, P. Główczyński, D. Tatar, P. Dębski

**Affiliations:** 1 Clinical Department of Psychiatry, Faculty of Medical Sciences, Medical University of Silesia, Tarnowskie Góry, Poland

## Abstract

**Introduction:**

For most people, gender identity is consistent with biological sex and such people are called cisgender. People in whom such a relationship does not occur or occurs to a lesser extent are referred to as gender non-conforming - and these include transgender, non-binary, agender and gender-fluid people. These groups are usually affected by minority stress, which, combined with the circumstances of the pandemic and war, may have led to mental disorders and sleep disorders in this population.

**Objectives:**

The aim of the study was to analyze the symptoms of anxiety, depression and insomnia in a group of Polish youth during the COVID-19 pandemic and the outbreak of the war in Ukraine, taking into consideration gender differences.

**Methods:**

The study involved 1621 secondary school students aged 14 to 19, the average age was 16.73±1.35, including 857 girls, 690 boys and 74 people who defined their gender as non-binary. A set of questionnaires for the Diagnosis of Depression in Children (CDI 2), the State-Trait Anxiety Inventory (STAI), the X-1 subscale, The Athens Insomnia Scale (AIS) and an original questionnaire of sociodemographic data were used in research.

**Results:**

Analyzing the results obtained in the study group, the respondents scored an average of 17.99 ±9.55pts in the assessment of depressive symptoms. After division into genders, the overall score was 19.69±9.40pts for girls, 15.03±8.68 for boys and 25.86±9.91 for non-binary people. The difference was statistically significant in all groups. In the anxiety symptoms assessment, the respondents scored an average of 46.92± 11.67pts. After division into genders, 49.21± 11.12pts for girls, 43.39 ± 11.47 for boys and 53.39 ± 10.41 for non-binary people. The difference was statistically significant in all groups. Analyzing the results obtained in AIS, the average score was 8.31±4.58pts, which allows to evaluate sleep onset as a norm. After dividing into groups, the results were 8.95±4.55pts for girls, 7.19±4.21 points for boys and 11.35±5.43 for non-binary people, the difference was statistically significant. Statistically significant positive correlations were found between the results obtained on the scale assessing depressive symptoms and anxiety symptoms and the results obtained on the AIS scale.

**Conclusions:**

Among the studied group of teenagers, the highest intensity of depressive symptoms is demonstrated by non-binary people, followed by females, and finally by males. Similar results were obtained in the assessment of anxiety symptoms. The non-binary group achieved results indicating sleep disorders, while the cisgender group’s results of sleep onset were borderline normal. Whatsmore, the greater the severity of depressive and anxiety disorders, the greater the sleep disorders in all study groups, regardless of gender.

**Disclosure of Interest:**

None Declared

